# Waterborne Transmission Driving the Prevalence of *Blastocystis* sp. in Los Ríos Region, Southern Chile

**DOI:** 10.3390/microorganisms13071549

**Published:** 2025-07-01

**Authors:** Daniel Sanhueza Teneo, Cedric B. Chesnais, Javiera Manzano, María Paz Moll, Analía Téllez, Guillermo Valenzuela-Nieto

**Affiliations:** 1 Instituto de Inmunología y Parasitología, Facultad de Medicina, Universidad Austral de Chile, Valdivia 5110566, Chile; javiera.manzano@alumnos.uach.cl (J.M.); maria.moll@uach.cl (M.P.M.); analia.tellezg@gmail.com (A.T.); 2 TransVIHMI, INSERM Unité, Institut de Recherche pour le Développement (IRD), Montpellier University, 34090 Montpellier, France; cedric.chesnais@ird.fr; 3 Facultad de Ciencias para el Cuidado de la Salud, Universidad San Sebastián, Valdivia 5090000, Chile; guillermo.valenzuela@uss.cl

**Keywords:** parasitology, public health, waterborne pathogens, *Blastocystis* sp., one health, environmental factors

## Abstract

Waterborne gastrointestinal infections remain a global health concern, with approximately 1.7 billion diarrhea-related illnesses annually attributable to protozoan parasites. These pathogens are transmitted through contaminated water and exhibit high resistance to chlorination, posing substantial challenges to effective water treatment. This study focused on the most prevalent intestinal parasites in the Los Ríos Region of Chile: *Blastocystis* sp., *Giardia duodenalis*, and *Entamoeba coli*. The objectives were to assess the prevalence of eukaryotic parasites in water samples—covering both drinking and recreational sources—to describe the circulating subtypes of *Blastocystis* sp. and to identify ecological factors associated with parasite presence. Water samples were analyzed using conventional PCR, next-generation sequencing (NGS) was employed for *Blastocystis* sp. subtype identification, and the environmental predictors were evaluated using a multivariable logistic regression model. A total of 132 water samples were analyzed, of which 15.2% were positive for *Blastocystis* sp. and 1.5% for *E. coli*, while no samples tested positive for *G. duodenalis*. We identified subtypes ST1–ST4 of *Blastocystis* sp., along with ST7, ST10, ST14, ST21, and ST23–ST26, the latter being reported for the first time in Chile. Ecological factors significantly associated with *Blastocystis* sp. presence included higher water temperature and greater rainfall at positive sites. Potable water was associated with significantly lower odds of *Blastocystis* sp. infection (aOR = 0.04, 95% CI: 0.00–0.87; *p* = 0.041), while precipitation increased infection odds by 3% per additional millimeter (aOR = 1.03, 95% CI: 1.00–1.06; *p* = 0.036). Greater distance to the nearest farmhouse was also significantly associated with reduced infection risk, suggesting that proximity to livestock environments may influence *Blastocystis* sp. transmission. These findings help explain the high prevalence of *Blastocystis* sp. observed in humans in the Los Ríos Region and highlight the pivotal role of ecological conditions in driving waterborne transmission. To our knowledge, this is the first environmental study in Chile to clearly demonstrate the association between human infection, environmental factors, and the transmission dynamics of *Blastocystis* sp.

## 1. Introduction

Nowadays, it is estimated that at least one in six people worldwide is affected by a parasitic disease. The global health burden is disproportionately concentrated in those countries with the greatest social and economic disadvantages [1]. According to the World Health Organization (WHO), waterborne gastrointestinal infections are among the leading causes of morbidity and mortality worldwide, with protozoan parasites responsible for 1.7 billion diarrheal illnesses and 842,000 deaths annually [2].

Intestinal protozoa are agents of widespread global distribution, whose transmission occurs mainly through the fecal-oral route through the consumption of water or food contaminated with their resistant stages, such as cysts or oocysts [3,4]. One of the most prevalent intestinal eukaryotic parasites in the world are *Blastocystis* sp., with prevalence rates close to 50% in developing countries and up to 20% in developed countries [5]; and *Giardia duodenalis*, with infection rates of up to 30% in developing countries and up to 8% in developed countries [6,7].

Chile has made a significant progress in the control of parasitic diseases. This can be attributed to the implementation of improvements in basic sanitation, drinking water supply, and sewage systems, as well as food control and the execution of strategies aimed at promoting literacy and health education [8].

However, there are limited current data on the prevalence of intestinal parasites in humans in the country. For example, Barra et al., in 2016 [9], determined the prevalence of intestinal parasites in schoolchildren in the commune of Puerto Montt. The prevalence of *Blastocystis* sp. was found to be up to 13%, *Endolimax nana* 12%, and *Entamoeba coli* 5%. Vidal et al., in 2010 [10], reported a significant increase in blastocystosis, with numbers up to 72.9% between 2005 and 2008. This coincides with a trend observed in the country, where blastocystosis is currently the most frequently diagnosed parasitosis through serial parasitological examination of stools (EPSD) [11].

There is also little data on intestinal parasites in the human population of the Los Ríos Region, southern Chile. In 1997, Torres et al. [12] analyzed stool samples from schoolchildren in different localities in the city of Valdivia and established prevalences of up to 51% for *Blastocystis* sp., 26.6% for *G. duodenalis*, 24% for *E. coli*, 18.4% for *E. nana,* 13.3% for the geohelminth *Trichuris trichiura*, and up to 7.2% for *Entamoeba histolytica*. More recently, Sanhueza-Teneo et al., in 2025 [13], described the prevalence of parasites in urban area of Valdivia, where *Blastocystis* sp. had 22% prevalence, followed by *E. coli* at 11.3% and *E. nana* at 3.5%. Finally, the most recent study of parasite prevalence in rural areas of Los Ríos Region establishes *Blastocystis* sp. as the most prevalent (29.2%), followed by *E. nana* (13.4%), *E. coli* (7.2%), and *G. duodenalis* (2.8%) [14].

Environmental factors, including suspended solids, oxygen levels, pH, organic matter, and temperature, play a crucial role in the viability and/or development of environmental resistance elements of parasitic stages [15,16]. Intestinal protozoa, such as *G. duodenalis* and *Cryptosporidium* spp., are excreted in the feces of infected humans and animals. These protozoa have been documented in numerous gastrointestinal disease outbreaks, predominantly in developing countries, having been transmitted by contaminated waters [17]. In addition, these pathogens possess a very low infectious dose. For example, for *G. duodenalis*, the intake of just 10 cysts is sufficient to cause infection in humans [7]. In terms of environmental persistence, parasites are characterized by their high resistance to chlorination and filtration, processes used in water treatment, which makes them a significant threat to public health [3]. As for *Blastocystis* sp. cysts, it has been reported that they can survive one month at room temperature and up to two months at 4 °C [18].

Studies of the genetic diversity of *Blastocystis* sp., has identified at least 40 subtypes (STs) [19]. Among these, ST1–ST8 have been reported in both humans and animals, while ST10–ST17 have been identified in non-human species [20]. Some subtypes are commonly found in particular hosts, such as ST3 in humans, ST5 in pigs, and ST7 in poultry [21]; however, no subtype is host-specific. Given the broad range of hosts, there have been findings of the same subtype of *Blastocystis* sp. in humans, their breeding animals, and in rivers adjacent to the housing sector [22].

In Chile, there are limited research in presence of parasites in environmental matrices. Suarez et al., in 2024 [23], identified the protozoa *Cryptosporidium parvum* using Ziehl–Neelsen staining and direct immunofluorescence assay (IFA) and *Blastocystis* sp. through conventional PCR in samples of mussels and treated wastewater (disinfected by chlorination) in a rural cove in the city of Concepción, Bío-Bío region. *C. parvum* and *Blastocystis* sp. were identified in 40% and 45% of the samples, respectively. Both parasites were also identified in the treated wastewater.

The interest area of this study includes the communes of Corral and Lago Ranco because they are among the communes with the highest prevalence of intestinal parasites in humans in Los Ríos Region. To that purpose, we implemented diagnostic techniques using endpoint PCR for three of the most prevalent parasites in this region: *Blastocystis* sp., *Giardia duodenalis*, and *Entamoeba coli* [14]. Finally, next-generation sequencing (NGS) technique were used to determine the subtypes of *Blastocystis* sp. in these two communities. The aim of this study was to determine the presence of eukaryotic parasites in water samples from the communes of Corral and Lago Ranco, in order to better understand the transmission pathways of these parasites in the region.

## 2. Material and Method

### 2.1. Characterization of the Study Area

The commune of Corral (39°53′ south latitude and 73°25′ west longitude) has an area of 767 km^2^ and is located approximately 20 km from the city of Valdivia, capital of the Los Ríos Region [24]. Its population is 5302, of which 65.7% live in urban areas and 34.3% in rural areas. In total, 54.3% of households lack access to basic services [25]. Corral is located at the mouth of the Valdivia River, and its territory includes coastal areas, forests, rivers, and mountain ranges [24]. The climate is temperate and rainy, with a cumulative rainfall of 1983 mm and an average annual temperature of 12.5 °C during the year 2024 [26].

On the other hand, the commune of Lago Ranco (40°19′00″ south latitude and 72°30′00″ west longitude), located in Ranco Province, Los Ríos Region, covers an area of 1763 km^2^ and has a population of 9896 inhabitants, of which 77.1% live in rural areas, and 36.7% reside in homes lacking basic services [27]. The hydrography of the commune consists of rivers, estuaries, lakes, and lagoons. Lake Ranco, the main body of water in the commune, covers an area of approximately 410 km^2^. Several rivers flow into the lake, including the Calcurrupe, Riñinahue, and Nilahue rivers [28]. The climate is temperate and rainy, with an annual precipitation of 1371 mm and an average temperature of 10.3 °C in 2024 [26].

The sampling points were georeferenced through Google Maps and subsequently imported into the QGIS Geographic Information System (v. 3.28) for the design of thematic cartographies.

### 2.2. Sampling

Sampling was conducted between August 2023 and June 2024, with the aim of collecting samples across all four seasons. In both communes of Corral and Lago Ranco, sampling sites were selected in areas with year-round geographic accessibility, ensuring comprehensive coverage of the study area.

According to international protocols (USEPA, WHO), the recommended volume for the detection of helminths and protozoa in water ranges from 1 to 10 L, depending on the type and quality of the sample [29]. However, due to logistical and operational constraints—such as limited time, transportation challenges, and filtration capacity—a sample volume of 3 L was established for stagnant and non-stagnant water bodies (estuaries, rivers, springs, ponds), and 2 L for rural drinking water (RW) samples, as these often required the cooperation of local residents, making the collection of larger volumes more difficult. Samples were collected in 1-L autoclaved glass bottles, protected from light with aluminum foil, and kept at 2–8 °C during transport. Upon arrival at the laboratory, they were stored at 4 °C until processing.

In Corral commune, 24 sampling points were established across five localities: 4 in the urban area of Corral, 5 in Huape, 3 in Chaihuín, 7 in Huiro, and 5 in Isla del Rey. A total of 69 water samples were collected in the study. Table 1 shows the number of samples collected at each site during each season.

In Lago Ranco, 19 sampling points were established across four localities: 2 in the city of Lago Ranco, 9 in Illahuapi, 4 in Riñinahue, and 4 in Calcurrupe. Over the entire sampling period, a total of 63 water samples were collected. Table 2 shows the number of samples collected in each locality during each season.

In total, 132 water samples were collected from both studied communes throughout the sampling period (69 water samples from Corral and 63 from Lago Ranco).

The number of samples collected during each season varied, due to logistical and environmental factors specific to each period, such as difficulties accessing certain areas due to adverse weather conditions or limited transportation availability. This variation was taken into account in the analysis and interpretation of the results.

### 2.3. Analysis of Abiotic Parameters in Water Samples

Abiotic parameters were measured in situ at the sampling points. Temperature (°C), pH, total dissolved solids (TDSs) (ppm), and electrical conductivity (EC) (mS/cm) were measured using the GrolineHI9814 instrument by Hanna Instruments^®^ (Padova, Italy).

### 2.4. Water Sample Processing

Sample processing was carried out based on the protocols described by Kumar et al. [30] and Sánchez et al. [31]. Samples were processed within 24 to 48 h of collection. Filtration of the samples was performed through disposable filtration systems connected to a vacuum filtration pump. The sample volume was completely filtered and then the filter was removed with sterilized scalpels and surgical forceps and deposited in a disposable Petri dish. The filter was cut into two equal halves; the first half was stored in the freezer at −20 °C until used for DNA extraction. The second half of the filter was used for observation by optical microscopy; for this, with a scalpel, the filter was cut into smaller segments that were deposited in a 15 mL centrifuge tube, after which they were washed with 13 mL of Tween 80^®^ 0.1% solution by vortex agitation. The washing liquid obtained was centrifuged at 3000 rpm for 10 min, the supernatant was discarded, and the sediment was subjected to the zinc sulfate flotation method described below.

### 2.5. Analysis of Water Samples by Microscopy

For the microscopic analysis of the water samples, the zinc sulfate flotation method modified from García in 2007 [32] was used. The sediment was resuspended with 1 to 2 mL of zinc sulfate (33 g of zinc sulfate in 67 mL of distilled water) and then zinc sulfate was added again until 11 mL of the tube was completed. It was centrifuged for two minutes at 1.800 rpm and zinc sulfate was added again to form a meniscus, in which, carefully, a 22 × 22 mm coverslip was placed and left to rest for 30 min. Each preparation was observed by light microscopy at 10× and 40×, and 100×. All preparations were performed in duplicate.

### 2.6. Extracting DNA from Water Samples

DNA extraction and storage were based by the results obtained by Hinlo et al. [33], who evaluated different conditions to maximize the recovery of environmental DNA from water samples. The DNA extraction kits used were QIAGEN^®^’ (Hilden, Germany)Dneasy Powersoil Pro-Kit and ThermoFisher (Waltham, MA, USA) Thermo Scientific GeneJET Genomic DNA Purification Kit.^®^ DNA extractions were performed following the manufacturer’s instructions.

### 2.7. PCR

PCR was performed using the ThermoScientific DreamTaq Green PCR Master Mix (2×) enzyme (ThermoFisher). The thermal profile of the amplification reactions included the following steps: initial denaturation at 94 °C for 5 min, followed by 35 cycles for each pair of primers with a denaturation at 94 °C for 1 min, annealing temperature at 50, 57, and 60 °C for *Blastocystis* sp., *G. duodenalis*, and *E. coli*, respectively, for 30 s and extension temperature of 72 °C for 1 min. Finally, final extension at 72 °C for 5 min.

As positive control, DNA samples extracted from human stool samples positive for *Blastocystis* sp., *G. duodenalis* and *E. coli* were used by light microscopy and endpoint PCR. Moreover, as a negative control, nuclease-free water was used.

The primers used for detection of each parasite are described in the Table 3.

### 2.8. Analysis of Subtypes of Blastocystis sp. by Using Next-Generation Sequencing

The positive samples for *Blastocystis* sp. by conventional PCR were sent to Austral-OMICS laboratory (Core facility at the Universidad Austral of Chile), but only fourteen positive samples were further analyzed using next-generation sequencing (NGS), targeting the 18rRNA gene. Basically, raw sequences obtained from Illumina MiSeq were quality-filtered and the Amplicon sequence variants (ASVs) were inferred using the *DADA2* package in R [37]. Taxonomic mapping was performed using the RDP Naive Bayesian Classifier algorithm and to identify ASVs belonging to *Blastocystis* sp., a phylogenetic tree was constructed using reference sequences from NCBI. Subtypes were determined by aligning representative sequences of each known *Blastocystis* sp. subtype with the *Blastocystis*-assigned ASVs. The relative abundance of ASVs was visualized using bar plots in R. A full description of the technique is provided in the Supplementary Materials.

### 2.9. Statistical Analyses

Physicochemical and environmental characteristics at the water collection site were used for statistical analysis to explain the presence of *Blastocystis* sp. only, because the number of positive samples for the other parasites was very low. Water type was categorized into three groups: standing water (ponds, etc.), non-standing water (rivers, streams, etc.), and drinking water. The four seasons were also included. All other variables were used as continuous quantitative variables: water temperature (°C), pH, TDS (ppm), EC (mS/cm), precipitation (mm), relative humidity (%), ambient temperature (°C), and distance from the water collection point to the nearest dwelling (km). The distance was measured using mapping tools from Google Maps.

Categorical variables were compared using the chi-square or Fisher’s exact test as appropriate. Quantitative variables were compared using the non-parametric Kruskal–Wallis test.

We fitted an adjusted logistic regression model including our variables, first examining multicollinearity via the variance inflation factor (VIF). Variables with VIF > 5 were considered to unduly imbalance the model and were excluded; the remaining variables were selected accordingly.

## 3. Results

### 3.1. Distribution of Samples with Parasites

**Figure 1:**
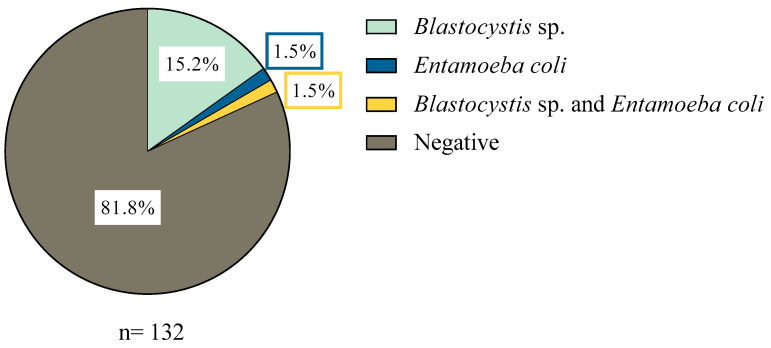
Positive samples with parasites in the communes of Lago Ranco and Corral.

In total, 132 water samples were analyzed by conventional PCR and microscopy. No parasites were observed by light microscopy and 24 samples were positive for at least one parasite by conventional PCR, which represents 18.2%. By parasite, 15.2% (20/132) of the samples had *Blastocystis* sp., 1.5% (2/132) had *Entamoeba coli*, and 1.5% (2/132) had both *Blastocystis* sp. and *E. coli*. (Figure 1). All samples were negative for *Giardia duodenalis*. Electrophoreses photographs for each commune are shown in Supplementary Materials.

**Figure 2:**
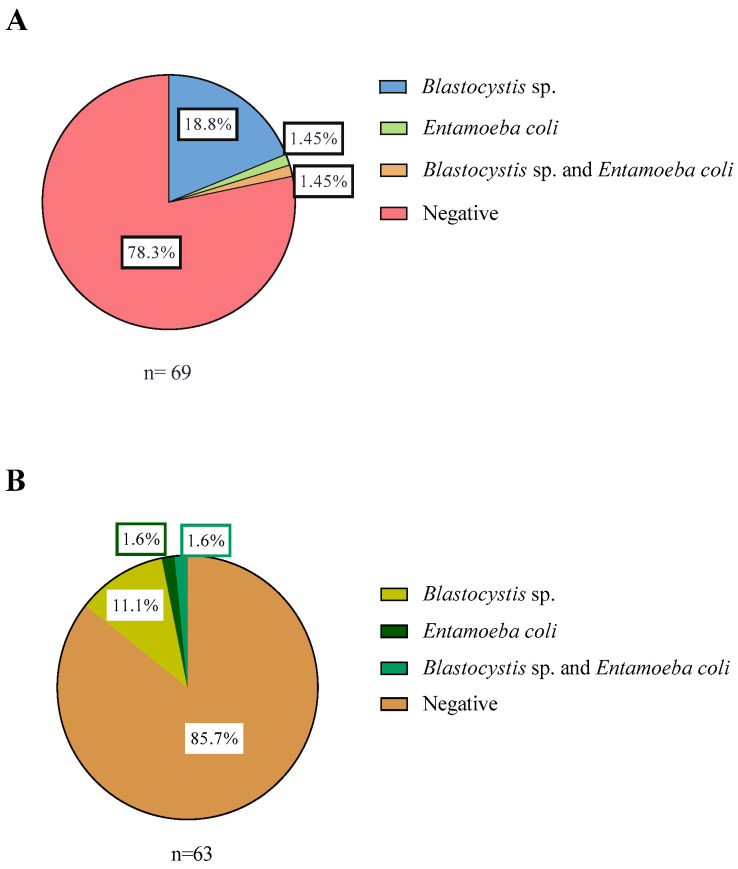
Percentage of samples with each parasite species described in water by conventional PCR: (**A**) Samples of Corral commune; (**B**) Samples of Lago Ranco commune.

For Corral, a total of 69 water samples were analyzed by conventional PCR. Here, 21% (15/69) was positive, where 18.8% (13/69) was positive for *Blastocystis* sp. only, 1.45% (1/69) for *E. coli*, and 1.45% (1/69) for both *Blastocystis* sp. and *E. coli* (Figure 2A). In Lago Ranco, a total of 63 water samples were analyzed by conventional PCR. No parasite DNA was detected in 85.7% (54/63) of the samples. *Blastocystis* sp. was detected in 11.1% (7/63), 1.6% (1/63) of the samples amplified for *E. coli*, and 1.6% (1/63) was positive for both *Blastocystis* sp. and *E. coli* (Figure 2B).

**Figure 3:**
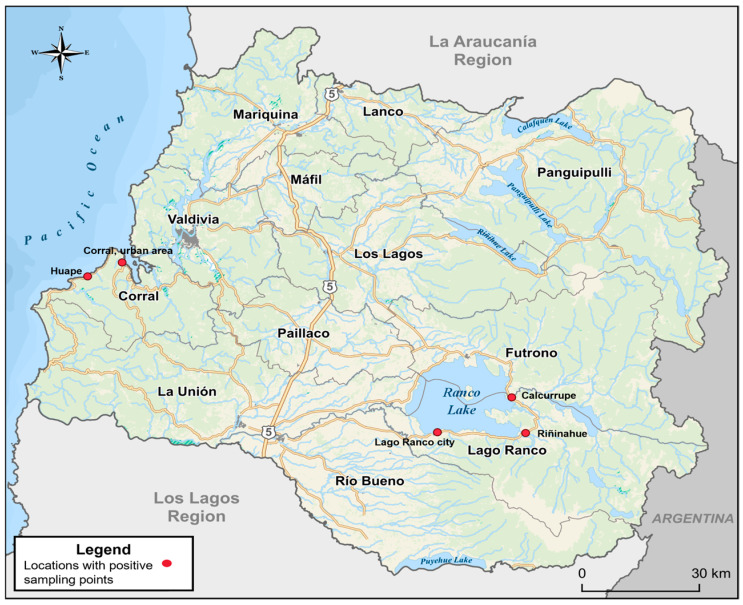
Geographic distribution of locations with positive sampling points in the communes of Corral and Lago Ranco.

The geographical distribution of the positive sampling points was Corral (urban area), Huape, Lago Ranco (urban area), Riñinahue, and Calcurrupe (Figure 3).

**Figure 4:**
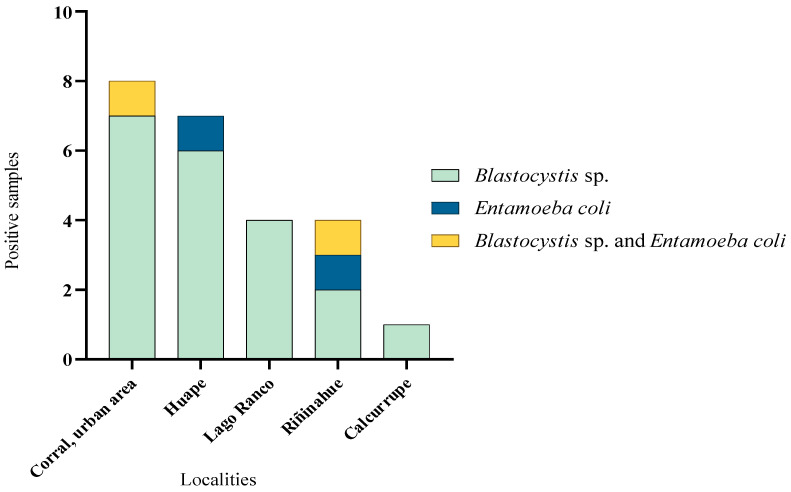
Number of water samples with parasites in all the positives sampling sites in Corral and Lago Ranco.

*Blastocystis* sp. was found in every positive site and the sampling point with the highest number of positive samples was the Corral urban area (Figure 4).

**Figure 5:**
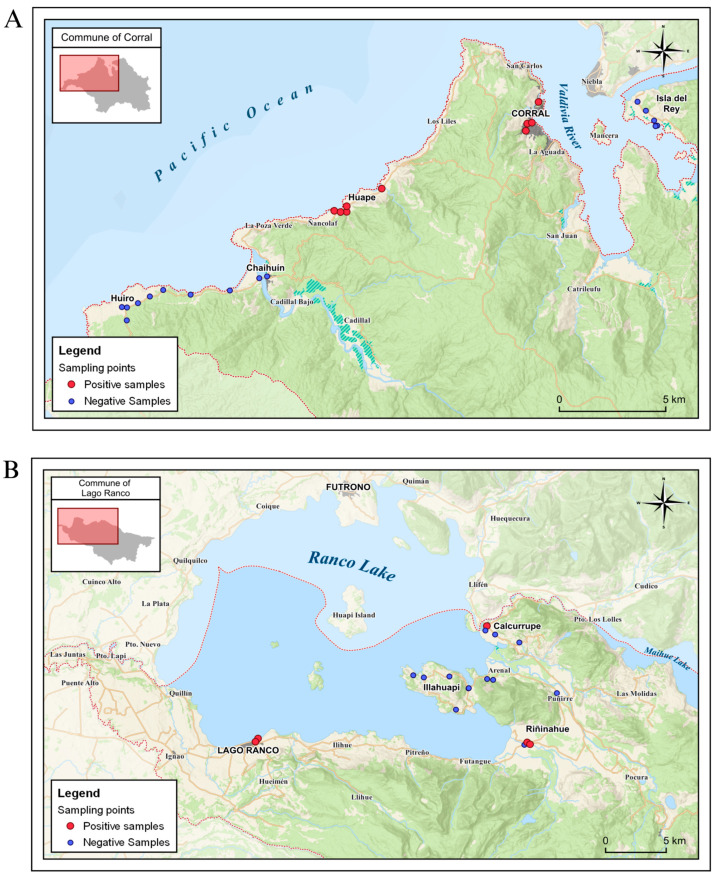
Geographical distribution of positive and negative samples: (**A**) Sampling points of Corral, commune; (**B**) Sampling points of Lago Ranco commune.

All of the positive samples of Corral came exclusively from the sectors of Corral (urban area) and Huape. In both locations, at least one positive sample was obtained at all sampling points (Figure 5A). The spatial distribution of the positive samples in the Lago Ranco commune shows that parasites were found in Lago Ranco (urban area), Riñinahue, and Calcurrupe (Figure 5B).

### 3.2. Seasonal Variation in Parasite Occurrence

**Figure 6:**
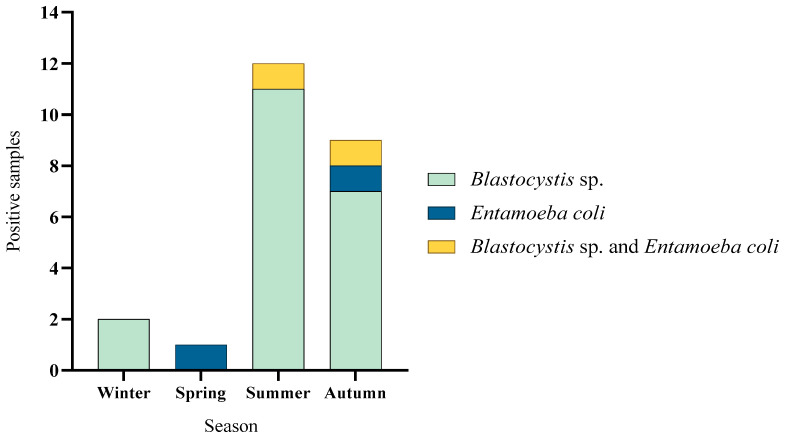
Seasonal distribution of positive samples with parasites in the communes of Lago Ranco and Corral.

Regarding the temporal distribution of parasites, these were found in all seasons of the year, with the highest number of positive samples being summer and autumn (*p* < 0.001). The highest diversity of parasites was found in autumn (Figure 6).

**Figure 7:**
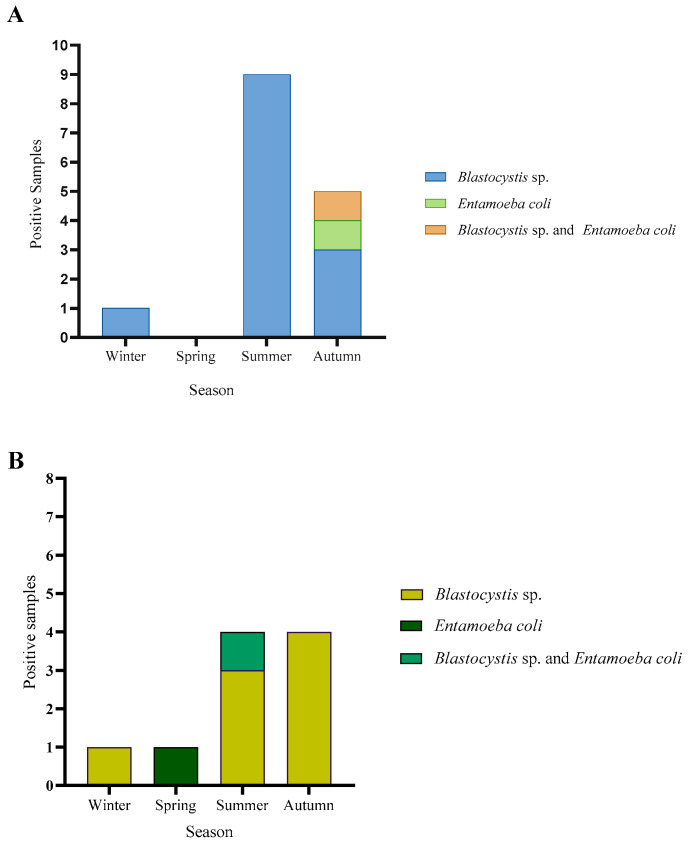
Seasonal distribution of positive samples with parasites: (**A**) Samples of Corral commune; (**B**) Samples of Lago Ranco commune.

The seasonal distribution in Corral showed that parasites were found in every season of the year, except spring. The highest number of positive samples were found in summer. The highest diversity of parasites was described in autumn, with *Blastocystis* sp. and *Entamoeba coli* found in this season. In winter and summer only, *Blastocystis* sp. was found in our samples (Figure 7A). In Lago Ranco, parasites were detected in every season of the year. *Blastocystis* sp. was present all through the year, except in spring, where only *E. coli* was detected. The highest number of positive samples was described in summer and autumn (Figure 7B).

### 3.3. Subtypes of Blastocystis sp.

Of the 14 positive samples—12 for Corral and 2 for Lago Ranco—for *Blastocystis* sp. that were successfully sequenced by Next-Generation Sequencing (NGS), 12 different subtypes were described in both communes. Sequences obtained from the analysis have been deposited in the GenBank database, following the accession number PRJNA1231441. The details of the proportion of the ASVs assigned for each commune is available in the Supplementary Materials.

**Figure 8:**
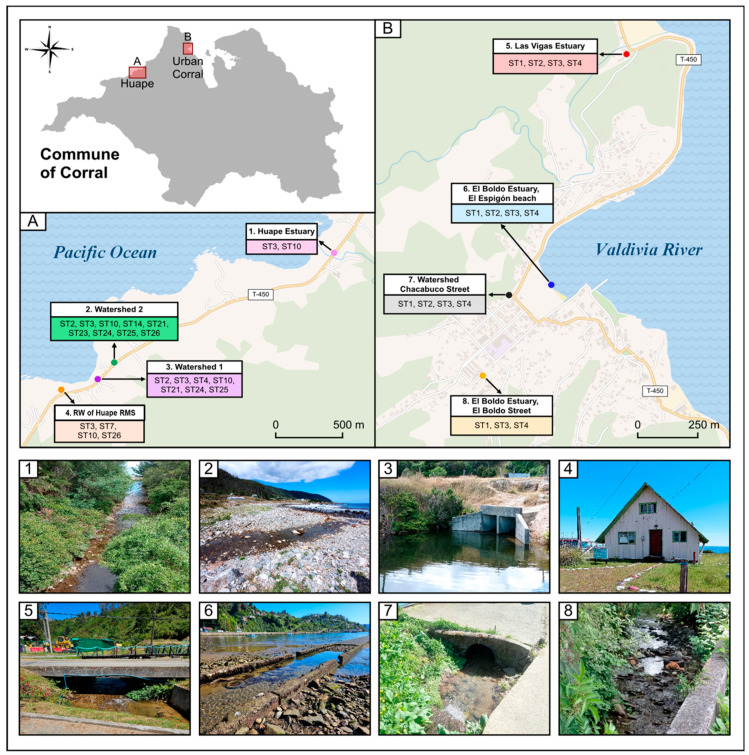
Description of spatial distribution of each subtype of *Blastocystis* sp. in Corral. (**A**) Subtypes described in the Huape sector and (**B**) subtypes described in Corral (Urban Area). Photos 1 to 8 show the exact sampling point in the commune of Corral (RW: Rural Drinking Water; RMS: Rural Medical Station).

In the commune of Corral, the subtypes described were ST1, ST2, ST3, ST4, ST7, ST10, ST14, ST21, ST23, ST24, ST25, and ST26. The details of the spatial distributions of each subtype are shown in Figure 8.

**Figure 9:**
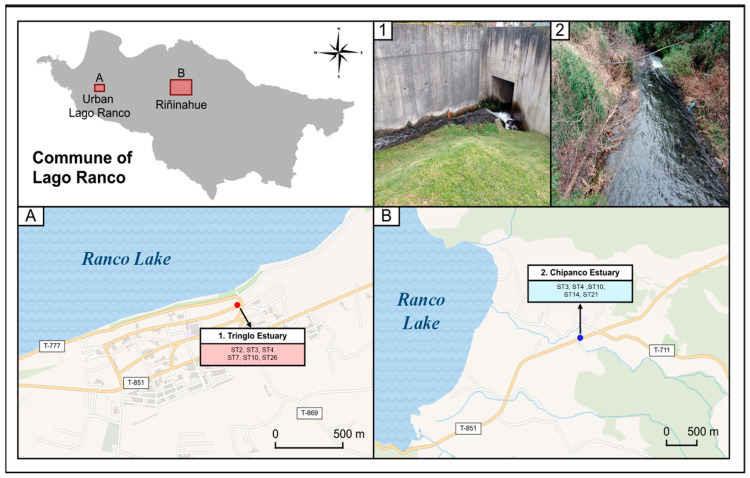
Description of spatial distribution of each subtype of *Blastocystis* sp. in Lago Ranco. (**A**) Subtypes described in Tringlo estuary sector and (**B**) subtypes described in Chipanco estuary. Photos 1 and 2 show the exact sampling points in the commune of Lago Ranco.

In Lago Ranco, the subtypes ST2, ST3, ST4, ST7, ST10, ST14, ST21, and ST26 were found. The details of the spatial distributions of each subtype are shown in Figure 9.

### 3.4. Environmental Factors Associated with Blastocystis sp. Presence

The descriptive analysis of water-collection site characteristics by *Blastocystis* sp. infection status is presented in Table 4. Overall, water temperature averaged 12.89 °C (±2.55) but was significantly higher among *Blastocystis* sp.-positive samples (13.97 ± 2.65 °C) compared with negatives (12.68 ± 2.49 °C; Mann–Whitney *p* = 0.021). Precipitation at collection sites also differed, with positives experiencing greater rainfall (41.48 ± 34.17mm) than negatives (28.44 ± 33.12mm; *p* = 0.002). Ambient air temperature was elevated in positive sites (11.97 ± 4.99 °C vs. 9.50 ± 3.38 °C; *p* = 0.034). Differences in pH, total dissolved solids (TDSs), electrical conductivity (EC), relative humidity, and density of farmhouses did not have statistical significance.

In our multivariable analysis, the season variable exhibited a high variance inflation factor (VIF > 10), reflecting substantial collinearity with other environmental predictors. After removing season from the model, all remaining VIF values fell below 5. The multivariable logistic regression model (Table 5) identified several environmental predictors of *Blastocystis* sp. presence. Water source emerged as a significant factor: compared with standing water, potable water was associated with a dramatically lower odds of *Blastocystis* sp. infection (aOR = 0.04, 95% CI 0.00–0.87, *p* = 0.041), whereas non-standing/river water showed no significant difference (aOR = 0.48, 95% CI 0.04–6.20, *p* = 0.572). Precipitation was positively associated with infection risk, with each additional millimeter increasing the odds by 3% (aOR = 1.03, 95% CI 1.00–1.06, *p* = 0.036). Distance to the nearest farmhouses also reached significance: greater distance strongly reduced infection odds (aOR ≈ 5.8 × 10^−9^, 95% CI 7.1 × 10^−16^–0.0472, *p* = 0.019), suggesting proximity to livestock environments may influence transmission. Other variables—including region (Lago Ranco vs. Corral), TDS, water temperature, pH, electrical conductivity, relative humidity, and ambient temperature—did not show statistically significant associations (*p* > 0.05), although relative humidity approached significance (aOR = 0.74, 95% CI 0.52–1.04, *p* = 0.079). These results indicate that water potability, rainfall, and proximity to farmhouses are key drivers of *Blastocystis* sp. contamination in this setting.

## 4. Discussion

PCR revealed *Blastocystis* sp. in 15.2% of samples, making it the most frequently detected parasite. This is the first study in Chile that describes the finding of *Blastocystis* sp. in recreational and drinking water. Similar prevalences of the parasite have been reported elsewhere: Khalifa et al., in 2014 [38], found 15.8% positivity in Egyptian pond water; Richard, in 2016 [39], found 27.7% in Malaysian drinking-water plants; and Karaman et al., in 2017 [40], found a prevalence of 20.6% in Turkish streams and drinking sources. Falcone et al., in 2024 [41]. reviewed 87 Latin America water-sampling studies (2000–2022) and noted *Blastocystis* sp. in 18.7% of them. Despite its public-health importance—recognized by the WHO as a parasite of concern in drinking water [42]—information on *Blastocystis* sp. in water remains scarce globally.

*Entamoeba coli* was detected in 1.5% of samples. This protozoan has been identified in wastewater samples in Spain [43], Iran [44], and Argentina [45]. Due to a similar mode of transmission, its public health implications in the region are comparable to those previously described for *Blastocystis* sp., with similar implications for fecal contamination monitoring.

The absence of *Giardia lamblia* in water samples, despite its well-established public health relevance [46], aligns with its low prevalence previously observed in the local human population [13]. This suggests a limited environmental circulation in the study area, in contrast to *Blastocystis* sp., which was frequently detected in both human and environmental samples. As for *Cryptosporidium* sp., although it is also a globally significant pathogen [46], a targeted search in water samples was not performed due to the lack of prior data on its circulation among the local population. In the present study, also no parasites were observed by light microscopy. This result is comparable to those reported by Ramos and Magtibay, 2020 [47], who likewise only found free-living organisms by light microscopy. The lack of parasitic elements by this method may reflect the limited sensitivity of light microscopy or the characteristics of the samples: (i) the small water volumes collected may reduce detection probability [29] and (ii) protozoan cysts and oocysts often occur at very low concentrations in water samples [48]

In terms of genetic diversity, the detection of multiple *Blastocystis* subtypes (STs) in both humans and animals—mammals, birds, and reptiles—underscores its zoonotic potential [49]. For example, ST7, commonly found in poultry, has been detected in individuals who work closely with these animals, and ST8 has been documented moving between non-human primates and their human caretakers [50]. Water, essential for all life, is therefore a key transmission route. In Nepal, Lee et al., in 2012 [22], found high ST4 prevalence simultaneously in villagers, and nearby rivers, strongly suggesting waterborne zoonotic transmission.

Globally, ST1–4 make up over 90% of human isolates, with ST3 the most common [49], and these subtypes also appear in animals and water. ST9, in contrast, was reported only in humans [42]. Our findings mirror this: ST1–4 were present in water samples from Lago Ranco and Corral, but ST9 was not. Animal-associated subtypes—ST7, ST10, ST12, ST14, and ST16—are less common in people [51] but ST7, ST10, and ST14 were found in our water samples of both communes. We also detected ST21 and ST23–26, typically linked to wild and domestic ruminants [52], though ST23 was first seen in 2021 in humans by Jinatham et al. [50] and ST26 in 2023 in Thai schoolchildren [53,54].

The results obtained in urban Corral are worth discussing compared to Huape. In the urban area of Corral, *Blastocystis* subtypes, commonly associated with humans (ST1–4), were mainly concentrated, whereas in Huape, a more rural area, a greater diversity of subtypes (ST7, ST10, ST14, ST21, ST23–26) was identified, frequently linked to domestic and wild animals. This difference could suggest a possible influence of zoonotic reservoirs and the existence of more diverse transmission routes in Huape, in contrast to the predominantly anthropogenic transmission in Corral. Meanwhile, in the city of Lago Ranco and Riñinahue, subtypes predominantly associated with humans were identified, along with subtypes linked to animals, in the same water source. This could indicate that these water bodies are exposed to a variety of hosts, suggesting the existence of more diverse transmission routes.

In Chile, human fecal studies have documented ST1–4 [14,55] and more recently ST6 [14]. Our work is the first to report ST1–4 in Chilean water, and to add ST7, ST10, ST14, ST21, and ST23–ST26 description. These environmental findings could help to explain the high 29% human *Blastocystis* sp. prevalence in the Los Ríos Region [14].

Although our descriptive and multivariable analyses confirmed the importance of natural factors—precipitation and water source—in *Blastocystis* sp. contamination, anthropogenic influences are equally critical. We found that infection risk decreases significantly as the distance between the sampling point and the nearest dwelling or farm increases. No significant differences were observed between regions, indicating that local characteristics—land use and livestock density—are the main drivers of fecal contamination. Nonetheless, it remains plausible that human population density further modulates risk: positive samples are clustered in more urbanized localities (Corral and Huape), where greater human density and infrastructural complexity can amplify sources of contamination [56,57]. In rural Chile, limited sanitation infrastructure and the proximity of latrines, livestock pens, and homes to watercourses facilitate the transfer of fecal matter into streams. Surface runoff during rainfall can rapidly carry cysts and oocysts to sampling points, while heavy rainfall may also dilute parasite concentrations [16]. These findings illustrate how anthropization—the alteration of natural landscapes by human settlement and land use—creates “hotspots” for *Blastocystis* sp. introduction and persistence. To mitigate transmission, targeted measures are needed: improving sanitation at water points, establishing buffer zones between latrines or animal enclosures and watercourses, and educating communities on safe water-handling practices.

### Study Limitations and Future Directions

The cross-sectional design limits temporal inference, and sample volumes constrained microscopy sensitivity. Future work should combine quantitative PCR with larger volume filtration and incorporate sanitary surveys to more precisely map fecal contamination sources. Moreover, integrating land-use GIS layers with high-resolution sanitation infrastructure maps could refine our understanding of anthropogenic risk factors.

## 5. Conclusions

Our findings confirm that *Blastocystis* sp. is a common waterborne parasite in southern Chile and highlight the dual importance of environmental conditions and human-driven landscape changes in its transmission. Enhanced sanitation measures and strategic water-point management are warranted to mitigate fecal pollution and protect community health.

## Figures and Tables

**Table 1:** Distribution of water samples collected in the Corral commune by season and sampling locality.

Localities	Winter	Spring	Summer	Autumn
Corral, urban area	2	4	4	4
Huape	4	5	5	5
Chaihuín	3	3	2	2
Huiro	2	7	6	6
Isla del Rey	-	5	-	-
Total samples	11	24	17	17
Total samples in Corral commune	69			

**Table 2:** Distribution of water samples collected in the Lago Ranco commune by season and sampling locality.

Localities	Winter	Spring	Summer	Autumn
Lago Ranco, city	2	2	2	2
Illahuapi	4	9	3	7
Riñinahue	4	4	4	4
Calcurrupe	4	4	4	4
Total samples	14	19	13	17
Total samples in Lago Ranco commune	63			

**Table 3:** Primers sequences used in this study.

Parasite	Gen	GenBank Code	Oligonucleotide Sequence 5′ to 3′	PCR Product Size	Reference
*Blastocystis* sp.	18s	XM_013038637.1	FS-GGAGGTAGTGACAATAAATC AS-GCTTTCGCACTTGTTCATC	538 bp	Santín et al., 2011 [34]
*Giardia duodenalis*	β-giardin	XM_001705373.1	FS-GAACGAGATCGAGGTCCG AS-CTCGACGAGCTTCGTGTT	455 bp	Maloney et al., 2020 [35]
*Entamoeba coli*	SSU Rrna	AF149915.1	FS-CTAAGCACAAAGTCCTAGTATGATG AS-CCTCATCGATTACACTCCCAG-AC	166 bp	Rattaprasert et al., 2021 [36]

**Table 4:** Mean and standard deviation of environmental variables according to *Blastocystis* sp. status.

Variable	Total Mean ± SD	*Blastocystis* − Mean ± SD	*Blastocystis* + Mean ± SD	*p*-Value (Mann–Whitney)
Temperature (°C)	12.89 ± 2.55	12.68 ± 2.49	13.97 ± 2.65	0.021
pH	6.94 ± 0.58	6.95 ± 0.60	6.91 ± 0.48	0.831
TDS (ppm)	163.18 ± 479.52	186.18 ± 522.53	48.18 ± 22.81	0.120
EC (mS/cm)	0.34 ± 0.96	0.39 ± 1.04	0.11 ± 0.05	0.096
Precipitation (mm)	30.61 ± 33.52	28.44 ± 33.12	41.48 ± 34.17	0.002
Relative Humidity (%)	84.85 ± 7.32	85.36 ± 7.22	82.59 ± 7.48	0.095
Ambient Temperature (°C)	10.03 ± 3.90	9.50 ± 3.38	11.97 ± 4.99	0.034
Farmhouses distance (km)	0.09 ± 0.19	0.10 ± 0.21	0.05 ± 0.04	0.707

**Table 5:** Logistic model explaining the presence of *Blastocystis* sp.

Variable	aOR (IC 95%)	*p*-Value
Lago Ranco region (Ref. Corral)	0.24 [0.02–2.39]	0.222
Type of water (Ref. standing water)		
No standing water/river	0.48 [0.04–6.20]	0.572
Potable water	0.04 [0.00–0.87]	0.041
TDS	1.02 [0.86–1.21]	0.831
Water temperature (°C)	1.15 [0.76–1.73]	0.510
Ph	0.35 [0.03–3.62]	0.379
EC (mS/cm)	8.91 × 10^−9^ [1.29 × 10^−47^–6.16 × 10^30^]	0.685
Precipitations (mm)	1.03 [1.00–1.06]	0.036
Relative humidity (%)	0.74 [0.52–1.04]	0.079
Ambient temperature (°C)	0.69 [0.39–1.23]	0.205
Farmhouses distance (km)	5.81 × 10^−9^ [7.14 × 10^−16^–0.0472]	0.019

## Data Availability

The original contributions presented in this study are included in the article/Supplementary Materials. Further inquiries can be directed to the corresponding author.

## Supplementary Materials

The following supporting information can be downloaded at: https://www.mdpi.com/article/10.3390/microorganisms13071549/s1: Title S1. Description of Next-generation Sequencing (NGS) technique; Figure S1:Electrophoresis of positive samples for *Blastocystis* sp. from Lago Ranco commune; Figure S2: Electrophoresis of positive samples for *Blastocystis* sp. from Corral commune; Figure S3: *Blastocystis* subtypes identified in Lago Ranco commune; Figure S4: Distribution of *Blastocystis* subtypes in Lago Ranco commune; Figure S5: *Blastocystis* subtypes identified in Corral commune; Figure S6: Distribution of *Blastocystis* subtypes in Corral commune. References [37,58,59,60,61] are cited in the Supplementary Materials.
