# Supplementary material for: Waterborne Transmission Driving the Prevalence of Blastocystis sp. in Los Ríos Region, Southern Chile
Source: Microorganisms. 2025 Jul 1;13(7):1549. doi: 10.3390/microorganisms13071549 (PMC12298328; doi:10.3390/microorganisms13071549)
Supplement: Supplementary file 1 [file microorganisms-13-01549-s001.zip › microorganisms-3687707-supplementary.pdf]

Table of contents

Title S1: Description of Next-generation Sequencing (NGS) technique 1

Figure S1. Electrophoresis of positive samples for *Blastocystis* sp. from Lago Ranco commune 2

Figure S2. Electrophoresis of positive samples for *Blastocystis* sp. from Corral commune 3

Figure S3. *Blastocystis* subtypes identified in Lago Ranco commune 4

Figure S4. Distribution of *Blastocystis* subtypes in Lago Ranco commune 5

Figure S5. *Blastocystis* subtypes identified in Corral commune 6

Figure S6. Distribution of *Blastocystis* subtypes in Corral commune 7

Title S1: Description of Next-generation Sequencing (NGS) technique

A methodology combining Trimomatic (v0.39;(1)) and PRINSEQ (v0.20.4;(2)) was used for sequence processing. First, Illumina adapter sequences were removed, and reads were filtered based on quality, retaining only those with an average Phred score ≥ Q30. Low-quality bases and ambiguous sequences (Ns) were also discarded. The Amplicon Sequence Variant (ASV) inference was performed using the DADA2 package in R (3), which includes error estimation by base, de-replication, ASV inference, paired read concatenation and chimera removal. Taxonomic mapping was conducted using the RDP Naive Bayesian Classifier algorithm (4) implemented in the DADA2 pipeline, along with the SILVA v138 database (5)

The ASV sequences that could not be taxonomically classified were aligned with ASVs previously assigned to *Blastocystis* sp. Unassigned ASVs identified as *Blastocystis* through phylogenetic analysis were then confirmed using BLAST against the NCBI database. Finally, to determine the subtype(s) of *Blastocystis* present(s) in the samples, representative sequences of each subtype available in the NCBI database were selected, and after aligned along with the *Blastocystis*-assigned ASVs. Finally, the relative abundance of ASVs in each sample was visualized using bar plots generated with the ggplot2 package in R.

References

1. Bolger AM, Lohse M, Usadel B. Trimmomatic: a flexible trimmer for Illumina sequence data. Bioinformatics. 2014 Aug 1;30(15):2114–20.

2. Schmieder R, Edwards R. Quality control and preprocessing of metagenomic datasets. Bioinformatics. 2011 Mar 15;27(6):863–4.

3. Callahan BJ, McMurdie PJ, Rosen MJ, Han AW, Johnson AJA, Holmes SP. DADA2: High-resolution sample inference from Illumina amplicon data. Nat Methods. 2016 Jul;13(7):581–3.

4. Wang Q, Garrity GM, Tiedje JM, Cole JR. Naive Bayesian classifier for rapid assignment of rRNA sequences into the new bacterial taxonomy. Appl Environ Microbiol. 2007 Aug;73(16):5261–7.

5. Quast C, Pruesse E, Yilmaz P, Gerken J, Schweer T, Yarza P, et al. The SILVA ribosomal RNA gene database project: improved data processing and web-based tools. Nucleic Acids Res. 2013 Jan;41(Database issue):D590-596.

Figure S1. Electrophoresis of positive samples for *Blastocystis* sp. from Lago Ranco commune

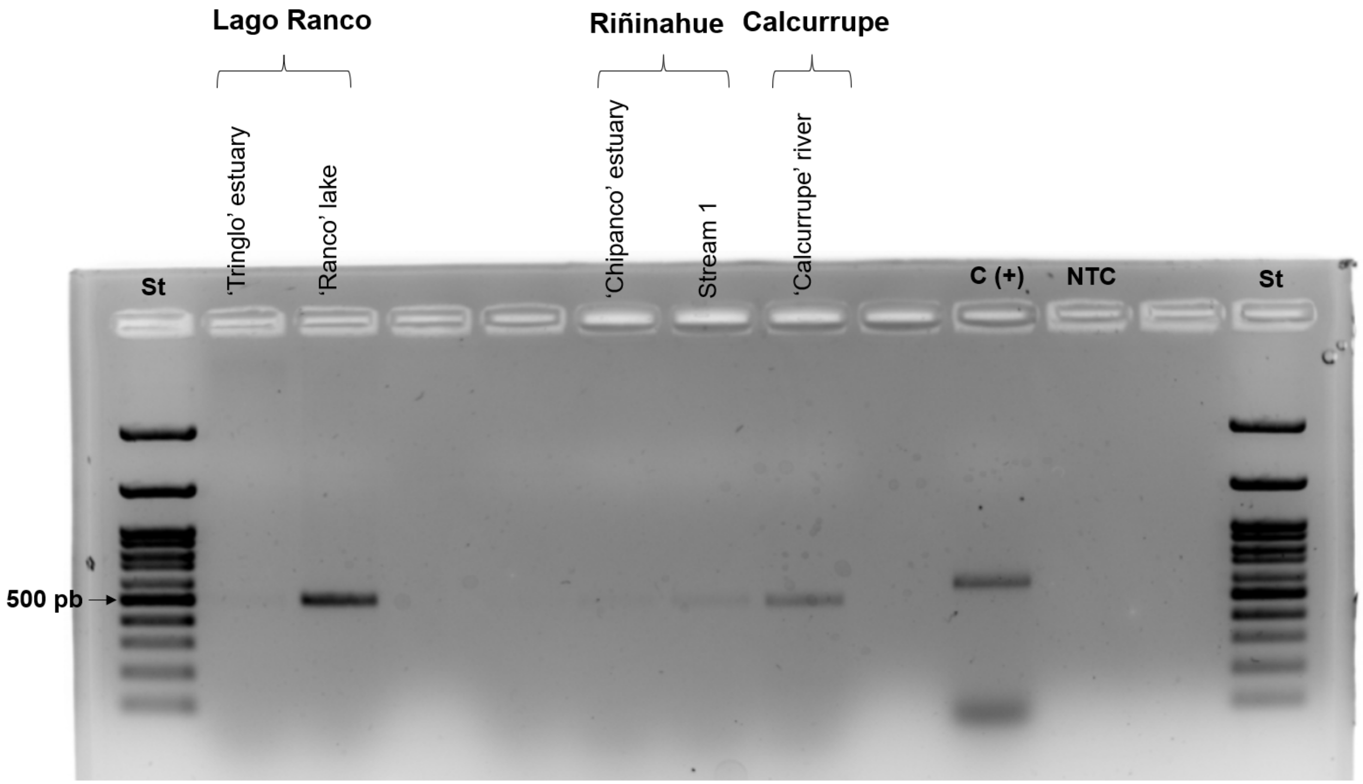

Figure 1. Positive samples for *Blastocystis* sp from Lago Ranco commune. Electrophoresis in 1.5% agarose gel stained with SafeView Plus 20000X. St. Lane corresponds to the DNA marker. Lane C+ corresponds to the positive control of the PCR, and the NTC lane corresponds to the negative control of the PCR

Figure S2. Electrophoresis of positive samples for *Blastocystis* sp. from Corral commune

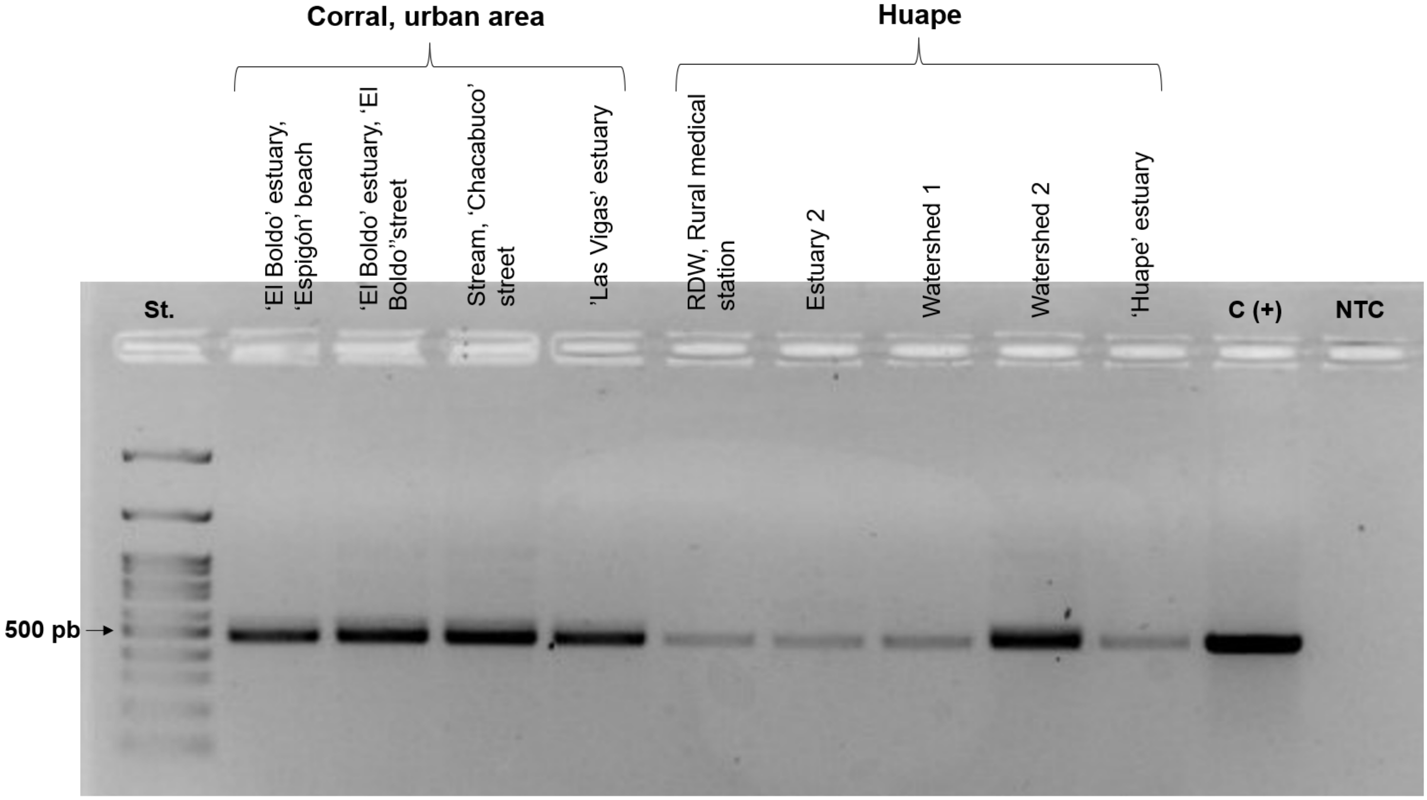

Figure 2. Positive samples for *Blastocystis* sp. from summer season of Corral commune. Electrophoresis in 1.5% agarose gel stained with SafeView Plus 20000X. St. Lane corresponds to the DNA marker. Lane C+ corresponds to the positive control of the PCR, and the NTC lane corresponds to the negative control of the PCR. RDW: Rural drinking water.

Figure S3. *Blastocystis* subtypes identified in Lago Ranco commune

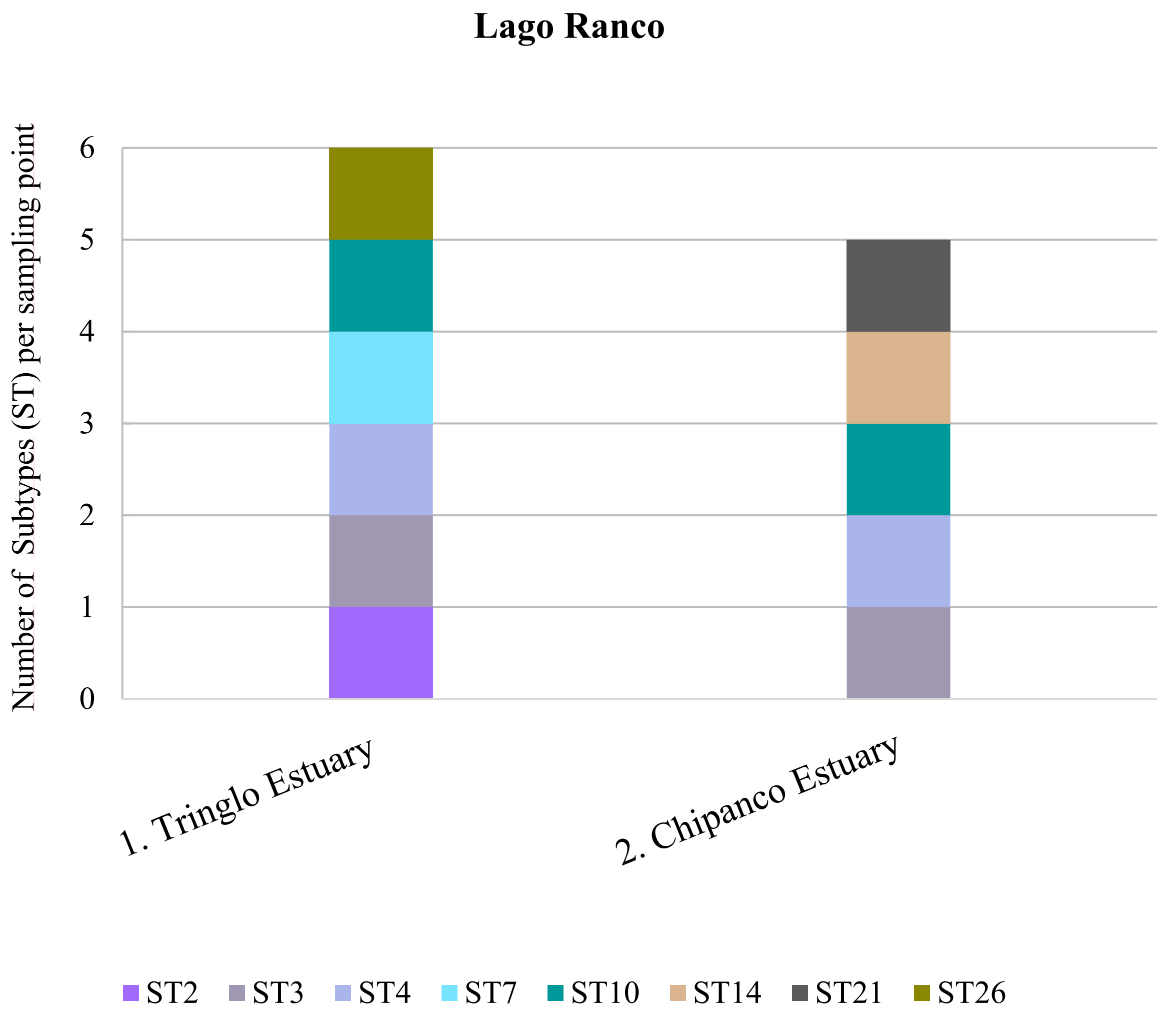

Figure 3. Number of *Blastocystis* Subtypes (ST) identified by NGS at sampling points in Lago Ranco commune.

Figure S4. Distribution of *Blastocystis* subtypes in Lago Ranco commune

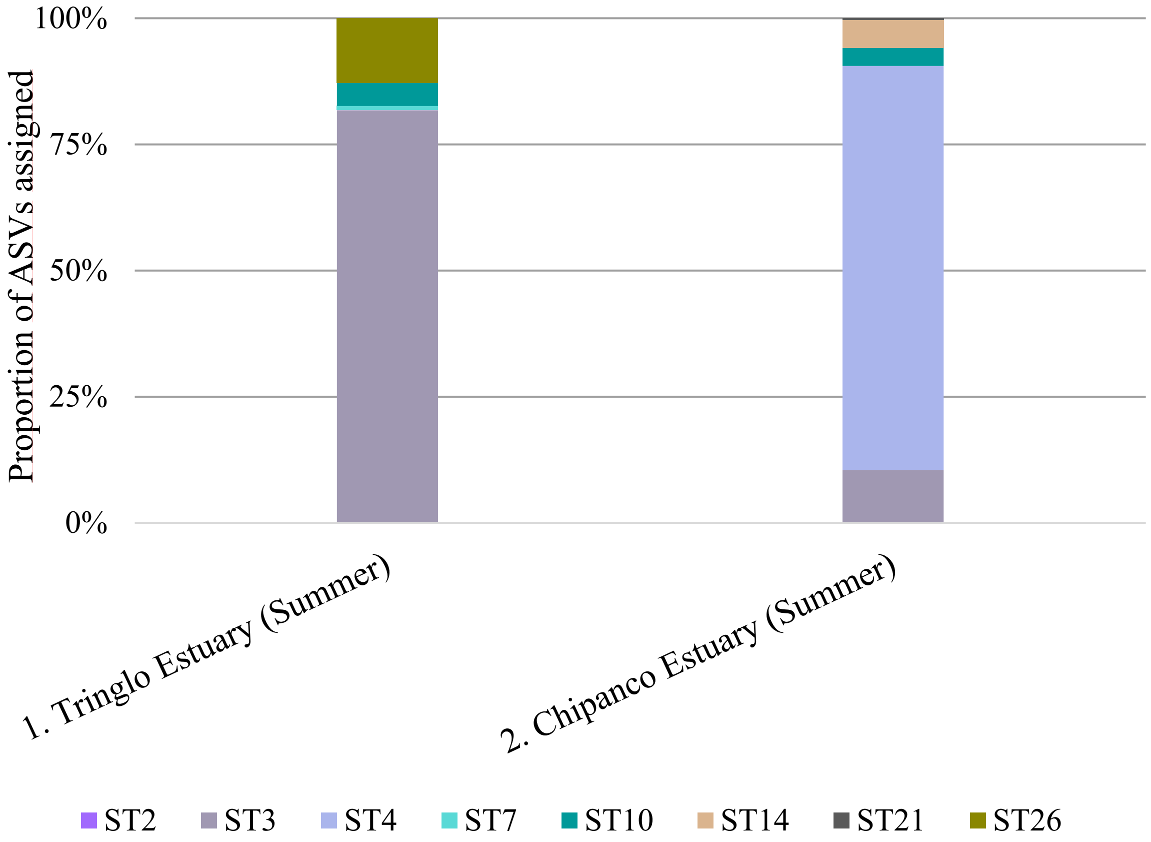

Figure 4. Distribution of *Blastocystis* Subtypes (ST) in water samples from Lago Ranco commune determined by Next Generation Sequencing (NGS).

Figure S5. *Blastocystis* subtypes identified in Corral commune

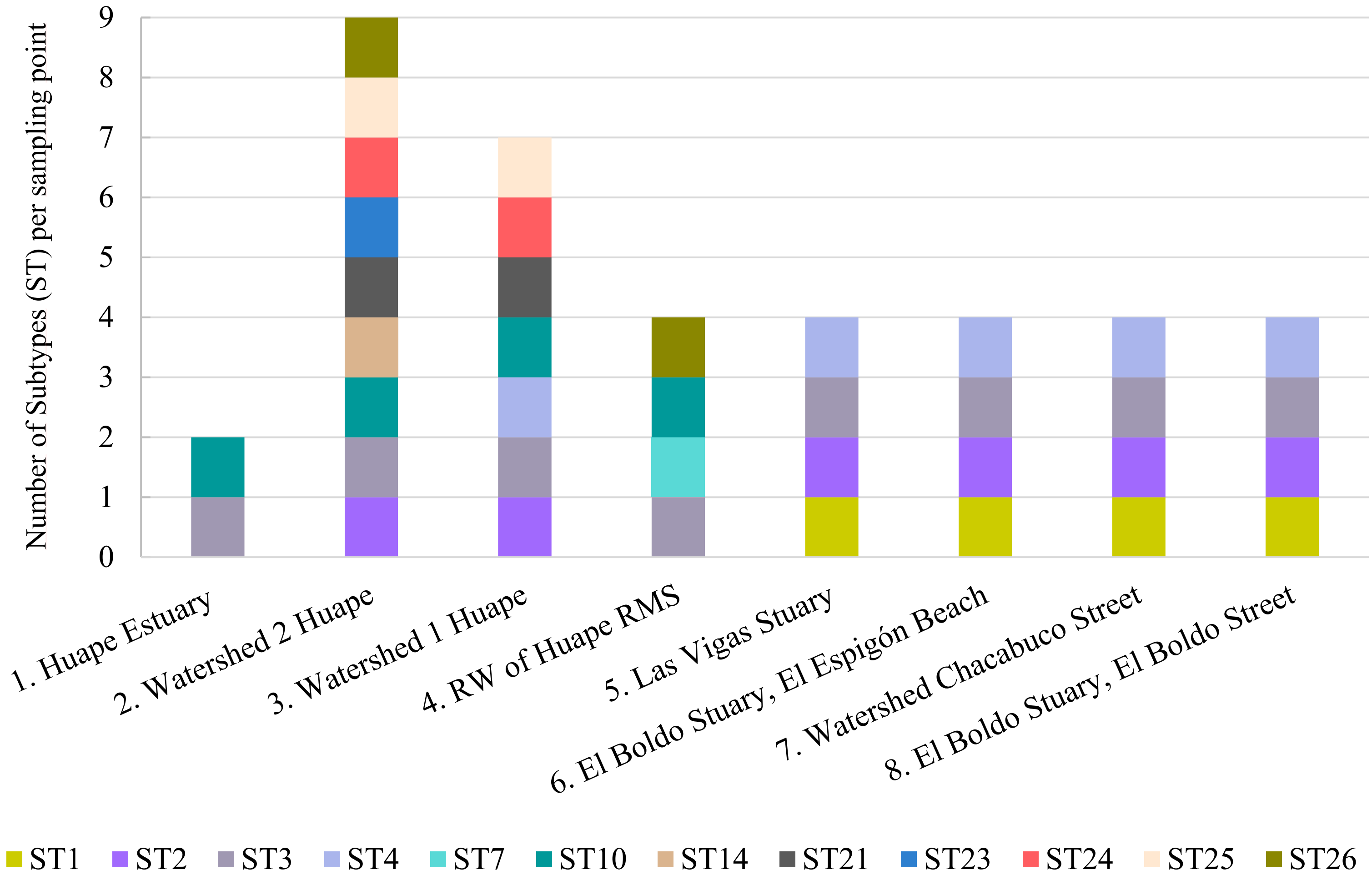

Figure 5. Number of *Blastocystis* Subtypes (ST) identified at sampling points in Corral commune.
(RW: Rural Drinking Water; RMS: Rural Medical Station).

Figure S6. Distribution of *Blastocystis* subtypes in Corral commune

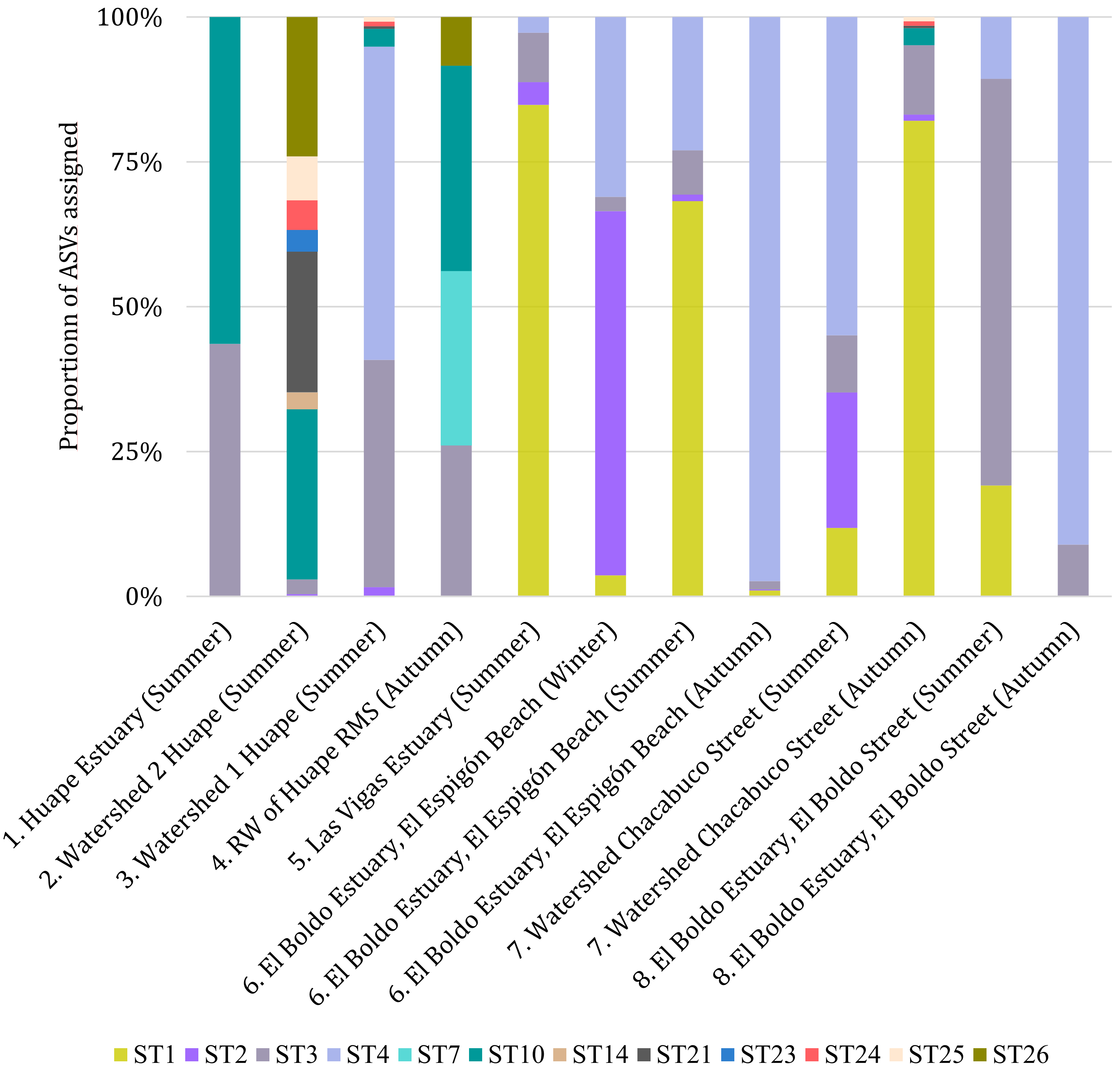

Figure 6. Distribution of *Blastocystis* Subtypes (ST) in water samples from Corral commune determined by Next Generation Sequencing (NGS) (RW: Rural Drinking Water; RMS: Rural Medical Station).
